# Balanced Fertilization Improves Crop Production and Soil Organic Carbon Sequestration in a Wheat–Maize Planting System in the North China Plain

**DOI:** 10.3390/plants14060838

**Published:** 2025-03-07

**Authors:** Huiyu Zhang, Hao Zhai, Ruixin Zan, Yuan Tian, Xiaofei Ma, Hutai Ji, Dingyi Zhang

**Affiliations:** 1 Institute of Wheat Research, Shanxi Agricultural University, Linfen 041000, Chinasxjihut@sxau.edu.cn (H.J.);; 2 College of Agriculture, Shanxi Agricultural University, Jinzhong 030800, China

**Keywords:** balanced fertilization, crop yield, SOC sequestration, soil nutrients and stoichiometry, labile SOC, microbial necromass C

## Abstract

Maintaining the long-term viability of a wheat–maize planting system, particularly the synchronous improvement of crop production and soil organic carbon (SOC) sequestration, is crucial for ensuring food security in the North China Plain. A field experiment in which wheat–maize was regarded as an integral fertilization unit was carried out in Shanxi Province, China, adopting a split-plot design with different distribution ratios of phosphorus (P) and potassium (K) fertilizer between wheat and maize seasons in the main plot (A) (a ratio of 3:0, A1; a ratio of 2:1, A2) and different application rates of pure nitrogen (N) during the entire wheat and maize growth period (B) (450 kg·ha^−1^, B1; 600 kg·ha^−1^, B2). Moreover, no fertilization was used in the entire wheat and maize growth period for the control (CK). The findings showed that A2B1 treatment led to the highest response, with an average wheat yield of 7.75 t·ha^−1^ and an average maize yield of 8.40 t·ha^−1^ over the last 9 years. The highest SOC content (15.13 g·kg^−1^), storage (34.20 t·ha^−1^), and sequestration (7.11 t·ha^−1^) were also observed under the A2B1 treatment. Both enhanced crop yield and SOC sequestration resulted from improvements in cumulative carbon (C) input, soil nutrients, and stoichiometry under the A2B1 treatment. It was confirmed that total N (TN), alkali-hydrolysable N (AN), available P (AP), available K (AK), and the ratios of C:K, N:K, and N:P had positive effects on crop yield through the labile components of SOC and on SOC sequestration through microbial necromass C. To conclude, our findings highlight the urgent need to optimize fertilizer management strategies to improve crop production and SOC sequestration in the North China Plain.

## 1. Introduction

Currently, the frequency of extreme weather events caused by the greenhouse effect is increasing globally, severely affecting human economies and life, particularly agricultural production and food security [1]. As the largest, most active, and most human-affected carbon (C) pool in the Earth’s terrestrial ecosystem [1], soil is considered the principal reason for the inter-annual variation in the concentration of carbon dioxide (CO_2_), a significant component of greenhouse gases (GHGs) [2]. Soil degradation in Chinese farmland is characterized by low levels of soil organic carbon (SOC) content and C input. These are also the primary factors limiting soil fertility and crop production improvement [3]. Therefore, increasing C input and SOC sequestration and reducing GHG emissions are essential pathways for achieving sustainable agricultural development in China.

Straw, a major by-product of agricultural production, contains abundant organic matter and nutrients [4]. Straw return can significantly increase SOC sequestration and reduce GHG emissions [5]. However, due to its relatively high C:N and C:P ratios, straw return can easily cause microbial nutrient limitations by affecting soil stoichiometry and triggering SOC mineralization [6], thereby impacting SOC storage [7]. A meta-analysis demonstrated that straw return combined with chemical fertilization can efficiently balance soil stoichiometry (C:N:P:K ratios), alleviate microbial nutrient constraints, and increase SOC content and crop output [8]. Song et al. [9] found that straw return combined with fertilization can alleviate nitrogen limitations for soil microorganisms during the winter wheat season, thereby reducing SOC mineralization and enhancing SOC storage. It is evident that straw return combined with different fertilizer practices significantly impacts SOC and crop output. There is a lot of research on the effects of straw return combined with different amounts or types of fertilizers on SOC. For example, a 12-year wheat–maize rotation experiment in the North China Plain showed that straw return combined with a higher nitrogen (N) application rate (>360 kg·ha^−1^) continuously increased SOC and microbial biomass carbon (MBC) content, significantly enhancing SOC storage, while straw return without fertilizer also increased SOC storage by 22.5% [10]. Field experiments conducted over 2 years in the semi-arid northwestern region indicated that a straw return of 9000 kg·ha^−1^ combined with 192 kg·ha^−1^ of N fertilizer significantly enhanced SOC storage by 14.2% compared to applying 240 kg·ha^−1^ of N fertilizer [11]. Additionally, studies based on combining optimized fertilization and straw returning have been published. These studies found that combining optimized fertilization (N, P, and K) and straw return significantly increased the sequestration of SOC at a soil depth of 0–20 cm when compared to applying only N fertilizer [12] or only N and P fertilizers [13]. Islam et al. [14] conducted a meta-analysis to evaluate the effect of straw return combined with different fertilizer practices, including balanced N, phosphorus (P), and potassium (K); unbalanced NP, NK, PK, or N fertilization; and no fertilization, on SOC storage in a wheat–maize cropping system, in which SOC storage showed the following conditions: balanced fertilization > unbalanced fertilization > no fertilization. In summary, the combination of straw return and chemical fertilizer can effectively balance the soil stoichiometric ratio and alleviate microbial nutrient limitations compared to straw return alone. However, the impact of this combination on SOC storage varies depending on the type and quantity of fertilizer used, and these results may also depend on differences in cropping systems, the natural climate, management practices, and initial soil characteristics [14].

Active components of SOC, such as particulate organic carbon (POC), labile organic carbon (LOC), dissolved organic carbon (DOC), and microbial biomass carbon (MBC), are more sensitive to farmland management practices and are usually used as early indicators to evaluate dynamic changes in SOC [15,16]. Microorganisms play an essential driving role in SOC transformation [17]. Soil microorganisms not only degrade SOC into CO_2_ [18] but also generate a significant amount of microbial necromass throughout the proliferation–death process, which directly contributes to SOC. Moreover, due to the structural differences in various microbial residues, the accumulation of different microbial residues can affect the stability of SOC [17,19]. Currently, the primary method for quantifying the content of microbial residues is quantifying microbial residue biomarkers, such as amino sugars [17,20]. Among these, glucosamine (GluN), which is primarily derived from fungal cell walls, and muramic acid (MurA), which is solely derived from bacterial cell walls, can estimate fungal necromass carbon (MNC) and bacterial necromass carbon (BNC), respectively, thereby allowing for the examination of the roles of bacteria and fungi in the process of SOC formation [21].

The wheat–maize cropping system, the largest and most significant planting pattern in China, produces 518 million tons of straw annually [14]. One of the main strategies for the Chinese 14th Five-Year Plan is to promote straw return with an overall utilization of 86%. Nevertheless, existing fertilization methods under straw return conditions in this cropping system primarily take into account the production of single-season crops, which results in problems such as the overuse of fertilizer, the acidification of the soil [7,8], and a limited increase in the balanced yield of double-season crops. Numerous studies have also examined the impacts of timing, quantity, and the combination of straw return and N fertilizer on the physical−chemical characteristics of soil, including SOC pools [4,5,22]. There is a lack of findings in the relevant literature on the effects of varying fertilization amounts for two-season crops in SOC pools when considering wheat–maize as an integral fertilization unit under straw-returning conditions. Accordingly, in this study, which was based on a 9-year field positioning experiment, wheat–maize was regarded as a fertilization unit, and different distribution ratios of P and K fertilizers and N fertilizer application rates were applied under full straw return conditions for two-season crops. The objectives were to (1) investigate the effects of different fertilizer practices on crop yield and SOC storage; (2) investigate the impact of different fertilizer practices on soil stoichiometry, active components of SOC, and microbial necromass C; and (3) analyze the correlations between the above indicators, particularly the factors affecting crop yield and indicators related to SOC, identifying field management practices that support the synergistic development of SOC sequestration and crop production in the wheat–maize cropping system in the North China Plain.

## 2. Results

### 2.1. Crop Yields and Cumulative C Inputs

**Figure 1:**
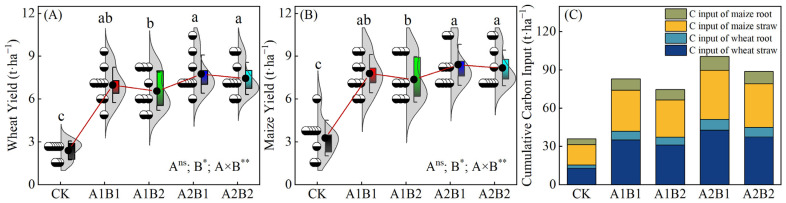
The yields of wheat (**A**), maize (**B**), and cumulative carbon input (**C**) under different fertilization treatments from 2014 to 2023: the 3:0 distribution ratio of phosphorus (P) and potassium (K) fertilizer between the wheat and maize seasons combined with 450 kg·ha^−1^ of pure nitrogen (N) during the entire growth period of wheat and maize (A1B1); the 3:0 distribution ratio of P and K fertilizer between the wheat and maize seasons combined with 600 kg·ha^−1^ of pure N during the entire growth period of wheat and maize (A1B2); the 2:1 distribution ratio of P and K fertilizers between the wheat and maize seasons combined with 450 kg·ha^−1^ of pure N during the entire growth period of wheat and maize (A2B1); the 2:1 distribution ratio of P and K fertilizers between the wheat and maize seasons combined with 600 kg·ha^−1^ of pure N during the entire growth period of wheat and maize (A2B2); no fertilization in the entire growth period of wheat and maize (CK). Different lowercase letters represent significant differences between different treatments based on Duncan’s multiple comparisons at *p* < 0.05. The upper and lower boundaries of the box plots represent the 75% and 25% quartiles, respectively. The upper and lower edges of the line in the whisker plot represent positive and negative error values, respectively. The black solid circle represents the average value of yield. The red line connects the average values of each treatment. *, **, and ‘ns’ indicate *p* < 0.05, *p* < 0.01, and *p* > 0.05, respectively.

The application of four fertilizer treatments resulted in a significant enhancement of wheat and maize yields in comparison with CK (Figure 1). The average wheat yields for 9 years under each fertilization treatment were 6.99 t·ha^−1^ for A1B1, 6.55 t·ha^−1^ for A1B2, 7.75 t·ha^−1^ for A2B1, and 7.44 t·ha^−1^ for A2B2, respectively. Similarly, the average maize yields were 7.78 t·ha^−1^ for A1B1, 7.38 t·ha^−1^ for A1B2, 8.40 t·ha^−1^ for A2B1, and 8.18 t·ha^−1^ for A2B2, respectively. Furthermore, A2B1 and A2B2 were found to be significantly higher than the A1B2 treatment, whereas no significant differences between A1B1 and these three treatments were observed. The results of the analysis of variance (ANOVA) demonstrate that the distribution ratio of P and K fertilizers in the wheat and maize seasons exerted no significant effects on crop yield, while the N application rate and the interaction significantly affected crop yield.

Additionally, cumulative carbon input under A1B1, A1B2, A2B1, and A2B2 treatments increased by 130%, 107%, 179%, and 146%, respectively, compared to CK.

### 2.2. SOC Content, Storage, and Sequestration

Soil bulk density (1.13~1.21 g·cm^−3^) under A1B2, A2B1, and A2B2 treatments was significantly lower compared with CK (Table 1). A2B1 significantly decreased soil bulk density by 7~9% more than A1B1 and A1B2, whereas it showed no significant difference compared with A2B2 treatments. Furthermore, SOC content, storage, and sequestration were significantly higher under A1B1, A2B1, and A2B2 treatments compared with CK, while no indicators under A1B2 were significantly different from CK. Moreover, the A2B1 treatment resulted in a significant enhancement of SOC content and storage, with increases ranging from 27% to 43% and from 16% to 33%, respectively, compared to the A1B1 and A1B2 treatments. However, no significant differences were observed between A2B1 and A2B2 treatments. A1B1, A2B1, and A2B2 treatments all demonstrated a tendency for SOC sequestration compared with the initial soil composition prior to the experiment, as evidenced by the order of A2B1 > A2B1 > A1B1, with a significant difference between them. Conversely, CK and A1B2 treatments exhibited a loss of SOC.

The ANOVA results demonstrated that the distribution ratio of P and K fertilizers in the wheat and maize seasons and the N application rate both significantly affected SOC content, storage, and sequestration, while the interaction had no significant effect on these indices. In addition, the distribution ratio of P and K fertilizers in the wheat and maize seasons may have had an indirect effect on soil bulk density.

### 2.3. Soil Nutrients

**Figure 2:**
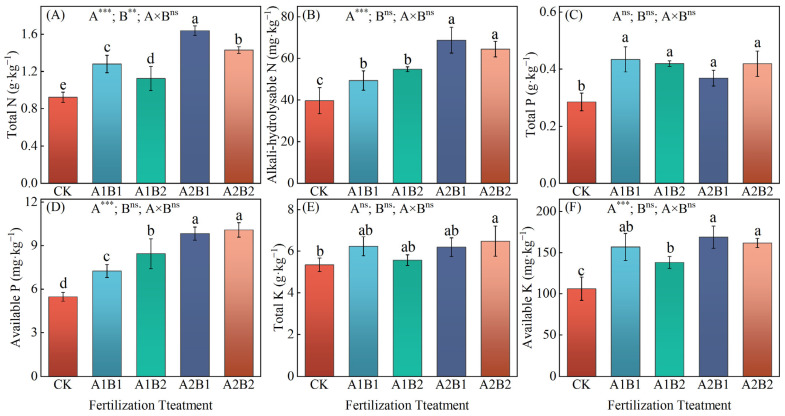
The contents of soil total N (**A**), alkali-hydrolysable N (**B**), total P (**C**), available P (**D**), total K (**E**), and available K (**F**) under different fertilization treatments. Different lowercase letters represent significant differences between different treatments based on Duncan’s multiple comparisons at *p* < 0.05. **, ***, and ‘ns’ indicate *p* < 0.01, *p* < 0.001, and *p* > 0.05, respectively.

Compared with CK, the TN (1.13~1.64 g·kg^−1^), TP (0.37~0.43 g·kg^−1^), AN (49.45~68.84 mg·kg^−1^), AP (7.26~10.09 mg·kg^−1^), and AK (138.35~169.04 mg·kg^−1^) contents were significantly enhanced under four fertilization treatments. For the TK content, A2B2 (6.49 g·kg^−1^) demonstrated a significantly higher value than CK. However, none of the differences observed between the other three fertilization treatments and these two treatments seemed to be statistically significant (Figure 2). The TN contents under the four fertilization treatments were ranked as follows: A2B1 > A2B2 > A1B1 > A1B2; significant differences were observed between the treatments (Figure 2A). A2B1 and A2B2 significantly increased AN, AP, and AK contents by 18~25%, 16~19%, and 17~22% (Figure 2B,D,F), respectively, compared with the A1B1 treatment, and significantly increased the AN and AP contents by 31~39% and 35~39%, respectively. However, there was no significant difference in the available nutrient content between A2B1 and A2B2.

The ANOVA results demonstrated that the distribution ratio of P and K fertilizers in the wheat and maize seasons significantly affected TN, AN, AP, and AK. Furthermore, the N application rate significantly affected TN, while the interaction had no significant effect on soil chemical properties.

### 2.4. Soil Stoichiometric Characteristics

In general, compared with CK, the C:P, C:K, N:P, and N:K ratios were significantly enhanced under the A2B1 treatment. Moreover, the N:K ratios under A1B1, A1B2, and A2B2 treatments were found to be significantly higher than those observed under CK (Table 2). The C:N ratio demonstrated no significant variation across the four fertilization treatments. The A2B1 treatment resulted in significantly higher C:P, C:K, N:P, and N:K ratios than the other fertilization treatments, while the A2B1 treatment resulted in a significantly lower P:K ratio than the A1B1 and A1B2 treatments. The ANOVA results demonstrate that the distribution ratio of P and K fertilizers in the wheat and maize seasons, as well as the N application rate, significantly affected the C:P, C:K, N:P, and N:K ratios. Furthermore, the interaction had significant effects on the C:P, C:K, and N:P ratios.

### 2.5. The Content of Active Components of SOC and Microbial Necromass C

**Figure 3:**
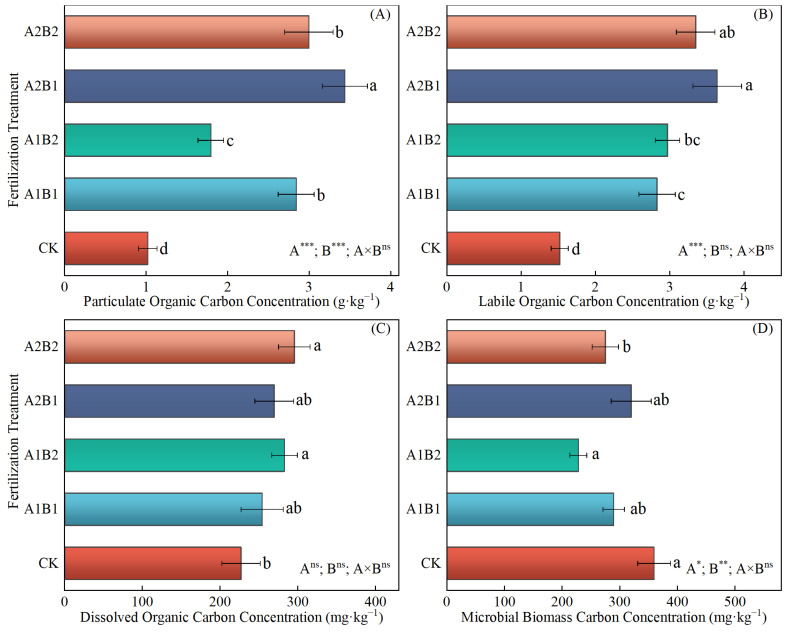
The concentrations of soil particulate organic carbon (**A**), labile organic carbon (**B**), dissolved organic carbon (**C**), and microbial biomass carbon (**D**) under different treatments. Different lowercase letters represent significant differences between different treatments based on Duncan’s multiple comparisons at *p* < 0.05. *, **, ***, and ‘ns’ indicate *p* < 0.05, *p* < 0.01, *p* < 0.001, and *p* > 0.05, respectively.

**Figure 4:**
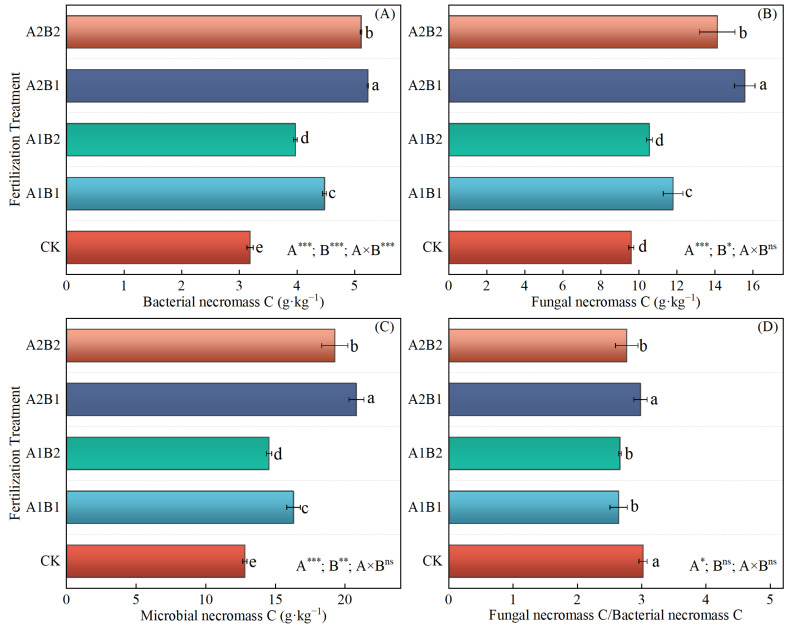
The contents of soil bacterial necromass C (**A**), fungal necromass C (**B**), microbial necromass C (**C**), and the ratio of fungal necromass C/bacterial necromass C (**D**) under different fertilization treatments. Different lowercase letters represent significant differences between different treatments based on Duncan’s multiple comparisons at *p* < 0.05. *, **, ***, and ‘ns’ indicate *p* < 0.05, *p* < 0.01, *p* < 0.001, and *p* > 0.05, respectively.

Compared with CK, the POC (1.79~3.44 g·kg^−1^) and LOC (2.83~3.64 g·kg^−1^) contents under all four fertilization treatments and the DOC content (282.77~295.39 mg·kg^−1^) under A1B2 and A2B2 treatments were found to be significantly increased. Conversely, the MBC content was lower than that of CK under the four fertilization treatments (Figure 3). No significant differences were observed in the DOC content among the four fertilization treatments (Figure 3C). The A2B1 treatment exhibited a 15~92% greater increase in POC content than the other fertilization treatments (Figure 3A) and a 23–29% greater increase in LOC content than the A1B1 and A1B2 treatments (Figure 3B). The MBC content under the A1B2 treatment was significantly higher than the A2B2 treatment, whereas it showed no significance with the A1B1 and A2B1 treatments (Figure 3D). Additionally, the BNC, FNC, and MNC levels showed a comparable trend under each treatment, with A2B1 > A2B2 > A1B1 > A2B2 > CK, where the differences among treatments were all significant (Figure 4). For the FNC/BNC ratio, a significantly higher value was observed under A2B1 the treatment than the other fertilization treatments, while no significant difference was observed with CK.

The ANOVA results demonstrate that the distribution ratio of P and K fertilizers in the wheat and maize seasons had a significant effect on POC, LOC, MBC, BNC, FNC, MNC, and the FNC/BNC ratio. Furthermore, the N application rate significantly affected POC, MBC, BNC, FNC, and MNC, while the interaction between these factors only had a significant effect on BNC.

### 2.6. Relationships Between Soil Chemical Nutrient Indicators, Crop Yield and SOC Indicators

**Figure 5:**
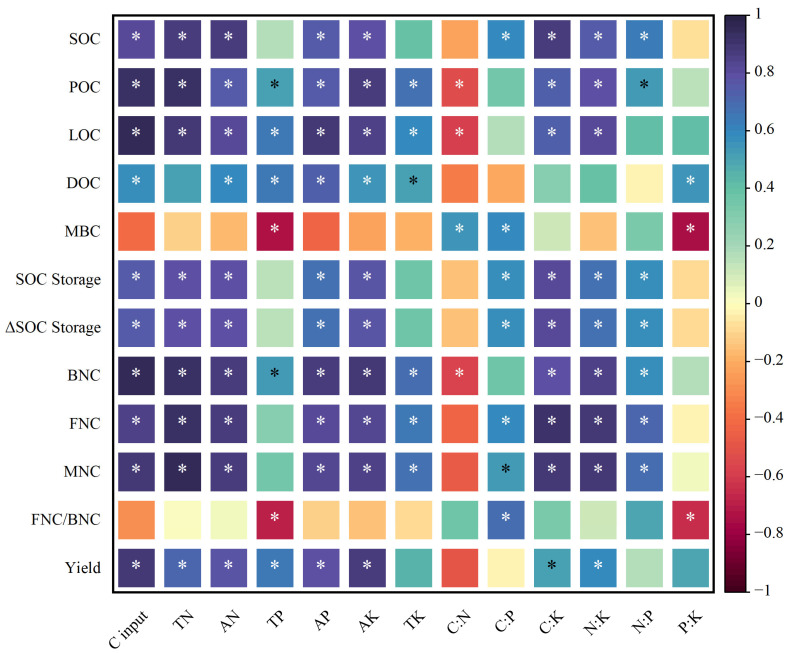
Pearson’s correlation coefficient between soil chemical nutrients, stoichiometry, related parameters of SOC, and annual crop yield. Total N (TN); alkali-hydrolysable N (AN); total P (TP); available P (AP); total K (TK); and available K (AK). * indicates *p* < 0.05.

The correlation analysis shows that crop yield was significantly and positively correlated with TN, AN, TP, AP, AK, C:K, and N:K (Figure 5). Furthermore, POC, LOC, BNC, FNC, and MNC were found to be significantly positively correlated with TN, AN, AP, TK, AK, C:K, and N:K, while POC, LOC, and BNC were significantly negatively correlated with C:N. Significant positive correlations were identified between DOC and AN, TP, AP, TK, AK, and P:K. MBC and the FNC/BNC ratio demonstrated significant positive correlations with C:P, while significant negative correlations were observed with TP and P:K. The SOC content, storage, and sequestration were found to be significantly and positively correlated with TN, AN, AP, AK, C:P, C:K, N:K, and N:P. In addition, a significant positive correlation was observed between the SOC content, storage, and sequestration, as well as TK, TP, AP, TK, and P:K. The ANOVA results demonstrate that the distribution ratio of P and K fertilizers in the wheat and maize seasons had significant effects on POC, LOC, MBC, BNC, FNC, MNC, and the FNC/BNC ratio. Furthermore, the N application rate significantly affected POC, MBC, BNC, FNC, and MNC, while the interaction between these factors only had a significant effect on BNC.

**Figure 6:**
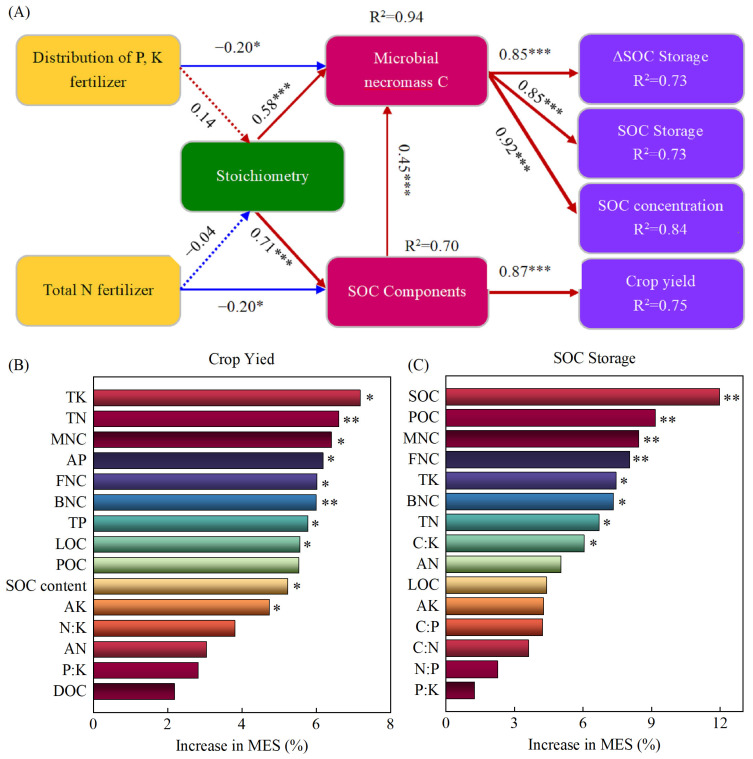
Structural equation (**A**) and random forest (**B**,**C**) models for annual crop yield and SOC sequestration affected by fertilization methods. In the structural equation model, the red and blue lines represent the positive and negative effects, respectively. The width of the line is proportional to the strength of factor loading. The number adjacent to the arrow line is a standardized coefficient that shows the variance explained by the variable. Solid and dotted lines indicate significant and non-significant effects, respectively. *, **, and *** indicate *p* < 0.05, *p* < 0.01, and *p* < 0.001, respectively.

Structural equation modeling (SEM) showed that the predictors accounted for 75% of the variation in crop yield, 84% of the variation in SOC content, and 73% of the variation in SOC storage and sequestration under the different fertilization methods (Figure 6A). The SOC components were found to be significantly influenced by the N application rate and stoichiometry with path coefficients of −0.20 and 0.71, respectively. The microbial necromass C (MNC) was found to be significantly influenced by the distribution of phosphorus and potassium fertilizers between the two seasons of wheat and maize, as well as stoichiometry with path coefficients of −0.20 and 0.57, respectively. Furthermore, crop yield was found to be significantly affected by SOC components with path coefficients of 0.87. The path coefficients of the effects of MNC on the SOC content, storage, and sequestration were 0.92, 0.85, and 0.85, respectively, indicating that optimizing fertilization methods and stoichiometry indirectly improved crop yield and soil carbon sequestration by regulating SOC components and MNC. The findings of this study indicate that random forest analysis accounted for 65.27% and 65.19% variation in crop yield and SOC sequestration, respectively. In the crop yield model (Figure 6B), TN and BNC were identified as superior predictors. In the SOC storage model (Figure 6C), the importance of soil properties decreased sequentially as SOC > POC > MNC > FNC > TK > BNC > TN > C:K.

## 3. Discussion

### 3.1. The Impacts of Different Fertilization Practices on SOC Storage and Its Regulating Factors

Soil nutrients are important regulatory factors in the decomposition of crop residues and SOC and, therefore, play a crucial role in controlling the dynamics of SOC in agricultural ecosystems [23]. Existing studies on the effects of nutrient availability on SOC under straw return conditions show contradictory results. Some studies have suggested that the long-term application of chemical fertilizers may not increase SOC sequestration in the long run after straw returns because high nutrient availability is conducive to the rapid decomposition of straw and SOC by reducing the C-nutrient stoichiometric imbalance [24,25,26]. Some studies have shown that the limitations of microbial biomass C increase under the condition of high nutrient availability, facilitating the increase in SOC storage over a long period of time [27,28]. In our study, SOC storage rates under the four fertilization treatments were improved to varying degrees compared with CK treatment under the condition of full straw return in a wheat and maize rotation planting system, consistent with the results of most studies [29,30,31], emphasizing that straw return destroyed the soil C and N balance, reduced the availability of soil N, and hindered N acquirement by microorganisms, thereby increasing microbial N restriction and affecting SOC accumulation [32].

In addition, SOC storage rates under the A2B1 treatment were significantly higher than those under the A1B1 and A1B2 treatments. Similar results have also been reported in previous studies [12,14,23], indicating that straw return combined with balanced fertilization is more conducive to SOC sequestration than unbalanced fertilization. The increase in SOC accumulation in a wheat and maize rotation planting system combined with full straw returning and balanced fertilization may be attributed to the following reasons: (i) Compared with unbalanced fertilization, the lower C:N ratio under balanced fertilization (Table 2) can effectively improve soil microenvironment, increase soil extracellular enzyme activity, improve microbial activity and nutrient balance, accelerate straw decomposition and nutrient release, and, thus, promote SOC sequestration [33,34]. At the same time, the higher C:N, C:P, and N:P ratios under balanced fertilization were conducive to SOC stability and sequestration [35]. (ii) Balanced fertilization contributes to the formation of above-ground dry matter and subsurface root biomass by improving nutrient availability for crop growth and, thus, has a positive feedback effect on SOC sequestration [36,37]. In our study, A2B1 significantly increased the C input of crop residues by 13–35% compared with the other three treatments and increased the organic matter returned to the soil, which entered the soil and directly served as the substrate of microorganisms [38], effectively increasing the content of active components of SOC in the soil, thereby promoting the accumulation of MNC and directly increasing SOC storage. Furthermore, the correlation results showed that the contents of TN, AN, AP, and AK and the ratios of C:K and N:K were significantly positively correlated with POC, LOC, and MNC, as well as the C input with active components of SOC, MNC, and SOC storage (Figure 5). Structural equation model results showed that stoichiometry had a direct positive effect on the active components of SOC and MNC contents, respectively, while it had an indirect effect on SOC storage through MNC (Figure 6A). The results of the random forest model demonstrate that the total explanation of SOC, POC, MNC, FNC, TK, BNC, TN, and C:K for SOC storage was 65% (Figure 6C). Therefore, our results further confirm that soil nutrients are important variables for regulating microbial nutrient limits. However, there was no significant difference in SOC storage between the A2B1 and A2B2 treatments, which may have been related to the fact that excessive N application may lead to soil acidification and the accumulation of toxic elements, inhibiting the growth of soil microorganisms and enzyme activities, and hindering the decomposition process of straw [39,40]. (iii) Higher SOC storage under balanced fertilization conditions was also attributed to improvements in leaf photosynthesis, biomass production, and K release, which are critical for crop growth [41]. An adequate K content can have positive effects on root elongation and biomass formation, as well as nutrient availability for crop growth [42]. More crop or root exudates are beneficial for increasing microbial activity, accelerating straw decomposition, and promoting SOC storage [36,37]. Therefore, balanced fertilization is essential for balancing soil nutrients and increasing SOC storage.

In our study, SOC sequestration under A2B1 treatment was 7.71 t·ha^−1^ compared with the initial state, equivalent to 28% of the relative change, which was significantly higher than those under A1B1 and A1B2 treatments and also confirmed the SOC sequestration advantage of the wheat maize rotation planting system compared with unbalanced fertilization or no fertilization. Compared with balanced fertilization, both unbalanced fertilization and no fertilization are less able to maintain native SOC storage, resulting in lower soil nutrient availability and the microbial fixation of N, which affects SOC storage [34,43]. Additionally, the relative change in SOC storage under A2B1 treatment was significantly higher than that under a wheat or maize monocropping system with 11~13% balanced fertilization, which may be attributed to the larger amount of returned straw in the wheat–maize planting system. Compared with 5~12% SOC sequestration efficiency under balanced fertilization conditions in semi-arid northwest China and the Northeast China Plain, the 7% SOC sequestration efficiency under A2B1 treatment in this study is different from that in the North China Plain, which may be attributed to differences in initial SOC levels and climatic conditions in different regions. For example, higher precipitation generally leads to better crop growth and higher C input, while low temperatures can lead to the slower decomposition of organic matter and decreased measurable SOC accumulation by significantly reducing microbial activity [38].

### 3.2. The Impacts of Different Fertilization Practices on Crop Yield and Its Regulating Factors

Maintaining crop production is an important part of the sustainable development of agricultural ecosystems, where crop yield is the most intuitive measurement indicator [44]. Fertilization is an effective way to enhance soil fertility and crop yield. Increased fertilization can increase crop yield within a certain range [45]. However, excessive or irrational fertilization reduces the efficiency of fertilizer utilization and causes various environmental problems [13]. The results of this study show that the annual crop yield of the wheat–maize planting system was significantly increased by fertilization under straw-returning conditions compared with no fertilization, and the largest increase was observed under the A2B1 treatment (Figure 1). Many studies have reported the positive effects of rational or balanced fertilization on crop yield [11,13,14,15]. This positive effect may be attributed to the following factors: (i) The improvement of soil properties, such as soil nutrients, stoichiometry, bulk density, etc., under balanced fertilization (Table 1, Figure 6B). The reduced soil bulk density under balanced fertilization [45] contributes to the transformation and release of soil nutrients and the better development of crop roots by increasing the porosity and aeration status of the soil, thus making it easier for crops to absorb and utilize nutrients in the soil. (ii) Balanced fertilization is beneficial to the formation of macroaggregates (>2 mm), and the role of aggregates in soil nutrient supply is altered by changing the distribution pattern of microbial enzyme activity in soil aggregates more than unbalanced fertilization or no fertilization [46]. Specifically, the microorganisms in microaggregates are easily restricted by C due to more native SOC being contained within it [46]. While macroaggregates showed higher N and P supplement capacities as they contain more active SOC, which increases the microbial community, total nutrients and available nutrients in macroaggregates are conductive to promoting the cycling of soil C, N, P and K, exerting a positive regulation effect on crop growth. (iii) Balanced fertilization reduced the C:N enzyme ratio and increased the N:P enzyme ratio, affecting microbial nutrient requirements by altering soil stoichiometry (high C:N ratio) [46]. More specifically, a higher SOC content stimulates microorganisms to produce more N-acquiring enzymes to satisfy their N requirements and maintain a microbial stoichiometric balance. In this study, the relatively high C:N ratio under A2B1 treatment was beneficial to the maintenance of soil stoichiometric balance. (iv) SOC and active components, as well as MNC, also had significant positive effects on annual crop yield (Figure 6B). A higher concentration of active SOC indicates active soil biochemical characteristics and a healthy soil C cycling system is conducive to maintaining sustainable agriculture productivity [45]. Active SOC can contribute to SOC accumulation and can improve soil fertility by having a positive effect on MNC (Figure 6A), thus promoting crop production. Overall, balanced fertilization can provide better growth conditions for subsequent crop growth by improving the physical, chemical, and biological properties of soil, especially the nutrient supply of soil macroaggregates. Therefore, to fully explore the mechanism of the crop yield increase effect under balanced fertilization, it will be necessary to consider the soil aggregate composition, as well as nutrient content, stoichiometry, and enzyme activity within it in future studies.

## 4. Materials and Methods

### 4.1. Description of Experimental Site

This field experiment was carried out at the Hancun Experimental Station of the Wheat Research Institute of Shanxi Agricultural University, which is located in Linfen City, Shanxi Province, China (36°06′25″ N, 111°30′55″ E), with a temperate continental semi-arid monsoon climate. The area has an average annual temperature of 13.1 °C, annual sunshine hours of about 2416 h, an average annual rainfall of 455 mm, and an annual evaporation rate of 2150 mm. The experimental field is irrigated plain land with calcareous brown soil, and the planting system is a “winter wheat–summer maize” rotational cropping system. The positioning experiment was started in 2014, and the initial values of soil fertility at a 0~20 cm depth were 10.34 g·kg^−1^ of SOC, 1.04 g·kg^−1^ of total N (TN), 42.76 mg·kg^−1^ of alkali-hydrolysable N (AN), 6.45 mg·kg^−1^ of available P (AP), and 123.67 mg·kg^−1^ of available K (AK).

### 4.2. Experimental Design

The short-term experiment began in October 2014, using the “winter wheat–summer maize” rotational planting system as the study carrier. The straw and root stubble of summer maize were crushed first after the previous maize was harvested and crushed again before land preparation. Then, the base fertilizer was applied, and the rotary tillage operation was implemented. Meanwhile, winter wheat was seeded using a rotary planter, with topdressing applied at the jointing stage. Following harvest, winter wheat straw was crushed and mulched on the surface. The summer maize was then sown directly, with topdressing applied at the flared opening and flowering−filling stages. A split-plot design was adopted. The main plot (A) had specific distribution ratios of P and K fertilizers between the wheat and maize seasons, including the ratio of 3:0 (A1) and the ratio of 2:1 (A2). The subplot (B) was the total N application rate during the entire growth period of wheat and maize, including 450 kg·ha^−1^ (B1) and 600 kg·ha^−1^ (B2) of pure N. Moreover, no fertilization in the entire growth period of wheat and maize was set as the control (CK). The experiment included 5 treatments (A1B1, A1B2, A2B1, A2B2, and CK) with 15 plots (3 m × 5 m = 15 m^2^), where three repetitions for each treatment were carried out. The types of N, P, and K fertilizers applied in the experiment were urea (N content 46.4%), diammonium phosphate (N~P_2_O_5_ content 18~46%, N~P content 18~20%), and potassium chloride (K_2_O content 60%, K content 50%). The total application rates of P_2_O_5_ and K_2_O were 180 kg·ha^−1^ and 135 kg·ha^−1^, respectively, and the ratio of N applied to winter wheat and summer maize was 5:5. In the winter wheat season, N fertilizer was applied during the sowing/jointing stage at a 7:3 ratio, and in the summer maize season, it was applied at the sowing/flare opening: the flowering−filling stage had a 3:5:2 ratio. The corresponding fertilizer application rates of each plot are shown in Table 3. The test varieties of winter wheat and summer maize were “Jimai 22” and “Dafeng 30”, with planting densities of 225 kg·ha^−1^ and 66,000 plants·ha^−1^, respectively. Other field management measurements were the same as those for local field production.

### 4.3. Sample Collection and Measurement Methods

At the time of wheat maturity, three representative areas of 1 m × 1 m were randomly selected in each treatment plot, and wheat plant samples were collected by cutting from the base of the plant with scissors. After the wheat plants were air-dried and threshed, the wheat kernels and straw were dried in an oven at 65 °C to a constant weight to determine the yields of the wheat kernel and straw weight. Following the maturation of the maize, 10 plants exhibiting uniform growth were selected at random from each treatment plot, with each plant sample collected in triplicate. Following this, the maize grains were separated, and the plant and kernels were air-dried. The maize grain yield and straw weight were then measured using a drying method similar to that described above.

In the course of the maize maturity period spanning 2020 to 2023, soil samples were collected from each plot at a depth of 0~20 cm using a stainless-steel cutting ring with a volume of 100 cm^3^, and this was repeated three times. The cutting rings containing soil samples were sealed and transported back to the laboratory. The external surfaces of the rings were wiped clean, and the solid covers were removed. The bottom of the mesh-covered cutting ring was then placed in a tray lined with absorbent gauze to absorb water for 12 h. During this period, water was added to ensure adequate hydration, taking care not to exceed the top edge of the mesh cover of the cutting ring. Once the soil sample in the cutting ring was saturated with water, it was dried in an oven at 105 °C to a constant mass and then weighed (M_1_). Finally, the soil sample was removed from the cutting ring and wiped clean from the cutting ring and mesh cover, and the mass (M_0_) of the cutting ring was weighed.

At the same stage, a 5 cm diameter soil auger was utilized to collect soil samples at a 0~20 cm depth within each plot, employing the five-point sampling methodology. The soil samples were subsequently transferred to the laboratory for the removal of stubble. A proportion of the samples was stored in a refrigerator at −4 °C to determine the DOC and MBC contents, while the residue was dried naturally and sieved to determine the soil chemical properties (TN, TP, TK, AN, AP, and AK contents); the SOC, POC, and LOC contents; and amino sugar content.

#### 4.3.1. Soil Chemical Nutrients

Air-dried soil samples were passed through a 0.25 mm sieve and weighed to determine the AN content, which was assessed by adopting the alkaline dissolution, diffusion, and absorption method. The determination of the TN, TP, AP, TK, and AK contents was conducted using an automatic Kjeldahl apparatus (Foss, Hilleroed, Denmark), a molybdenum antimony colorimetry with a UV-Vis Spectrophotometer (Thermo Evolution 60S, Waltham, MA, USA) and the flame photometric method with a flame photometer (Thermo M5, Waltham, MA, USA) [47].

#### 4.3.2. SOC and Its Components Content

Air-dried soil samples previously passed through a 0.25 mm sieve were weighed for SOC and LOC determination. The SOC was determined via the K_2_Cr_2_O_7_-H_2_SO_4_ oxidation method, and the LOC was determined via the KMnO_4_ oxidation method. Air-dried soil samples passed through a 2 mm sieve were extracted with a sodium hexametaphosphate solution at a concentration of 5 g·L^−1^, and then POC was determined via the K_2_Cr_2_O_7_-H_2_SO_4_ oxidation method. The determination of DOC was achieved by applying the Mn (III)–pyrophosphate complex eclipsing method using a UV-Vis Spectrophotometer (Thermo Evolution 60S, Waltham, MA, USA). The MBC was determined using the chloroform fumigation extraction method with a TOC analyzer (Thermo ICP 6000, Waltham, MA, USA) [36].

#### 4.3.3. Amino Sugar Content

The amino sugar content of the soil was determined via the gas chromatography method [20]. Specifically, following the weighing of a soil sample, 6 mol·L^−1^ of HCl was added, hydrolyzed at 105 °C for 8 h, and filtered. The filtrate was then dried and adjusted to a pH of 6.6~6.8, following which it was subjected to centrifugation. Thereafter, anhydrous methanol was added after the precipitate was removed. The resultant dissolved layer was transferred to the derivative bottle and dried with high-purity N for freeze-drying. The sample was then dried with derivative reagents, dissolved with dichloromethane, and then added with an HCl vortex to remove the upper inorganic phase. The remaining organic phase was then subjected to freeze-drying using high-purity N. The residues were dissolved in a mixture of ethyl acetate and n-hexane (1:1) and analyzed via gas chromatography (Vanquish Core, Waltham, MA, USA). The contents of amino sugar were calculated via the internal standard method.

### 4.4. Calculation of C Input of Crop Residue, SOC Storage, and Sequestration

#### 4.4.1. Carbon (C) Input of Straw Residue

C input was estimated according to Equations (1)~(3) [48]:

Cinput = Cinput_straw_ + Cinput_root_ (1)

Cinput_straw_ = Y_wheat_ × 0.378 + Y_maize_ × 0.430 (2)

Cinput_root_ = Y_wheat_ × 0.378 × 0.20 + Y_maize_ × 0.430 × 0.28 (3)

where Cinput, Cinput_straw_, and Cinput_root_ denote the total C input of the crop residue, crop straw and crop root, respectively; Y_wheat_ and Y_maize_ denote the amounts of wheat and maize straw (t·ha^−1^), respectively; 0.378 and 0.430 denote the conversion coefficients of C content in wheat and maize straw, respectively; and 0.20 and 0.28 are the root/shoot ratios of wheat and maize, respectively.

#### 4.4.2. Soil Bulk Density, SOC Storage and Sequestration

Soil bulk density can be calculated using Equation (4):

BD = (M_1_ − M_0_)/100 (4)

where BD is the soil bulk density (g·cm^−3^), M1 is the mass of the cutting ring and dry soil (g), M0 is the mass of the cutting ring (g), and 100 is the volume of the cutting ring (cm^3^).

SOC storage was calculated adopting Equation (5):

SOC storage = (C_SOC_ × BD × D)/10 (5)

where C_SOC_ is the SOC content (g·kg^−1^), D is the thickness of the soil layer, and 10 represents the conversion coefficients.

SOC sequestration was estimated by Equation (6):

ΔSOC Storage = SOC Storage_nyear_ − SOC Storage_0_ (6)

where ΔSOC Storage is SOC sequestration (t·ha^−1^), SOC Storagenyear is the SOC storage after n years of fertilization management (t·ha^−1^), and SOC Storage0 is the SOC storage before the experiment (t·ha^−1^).

#### 4.4.3. Microbial Necromass Carbon (MNC)

Microbial necromass carbon was calculated using Equations (7)~(9) [21]:

BNC = MurA × 31.3 (7)

FNC = (GlcN − 1.16 × MurA) × 10.8 (8)

MNC = BNC + FNC (9)

where MurA is the cell acid content (mg·kg^−1^), GlcN is the glucosamine content (mg·kg^−1^), BNC is bacterial necromass carbon, FNC is fungal necromass carbon, MNC is total microbial necromass carbon, 31.3 is the conversion coefficient of cell wall acid-to-bacterial necromass carbon, and 10.8 is the conversion coefficient of fungal glucosamine-to-fungal necromass carbon.

### 4.5. Statistical Analysis

The differences between different treatments were examined using a one-way analysis of variance, followed by Duncan’s multiple comparison test at *p* < 0.05. Statistical analyses were performed using SPSS 27.0 software (SPSS Inc., Chicago, IL, USA). Pearson correlation analysis was performed using Origin 2022 (Origin Lab Inc., Northampton, MA, USA) to identify significant relationships between indicators. The figures were generated using Origin 2022 (Origin Lab Inc., Northampton, MA, USA). A structural equation model (SEM) was constructed using Smart-PLS 4.0 to explore the influence of fertilization methods and stoichiometry on crop yield, SOC content, storage, and sequestration. In SEM analysis, fertilization methods were set up with three levels of categorical variables based on the fertilizer application ratio or rate as follows: 0 (CK), 3 (A1), 2 (A2), 450 (B1), and 600 (B2). To quantify the contribution of each explanatory variable to variations in crop yield and SOC storage, a random forest analysis was performed to evaluate the most important factors using the R “randomForest” package and the “rfPermute” package in the R Statistical Environment (Version 4.3.2, R Core Team).

## 5. Conclusions

Overall, in a wheat–maize planting system with full straw return, fertilization improved the average crop yield over the past 9 years, as well as the SOC content and storage; however, there were differences in yield and SOC effects between the fertilization treatments. A2B1 treatment resulted in the highest average yields of wheat (7.75 t·ha^−1^) and maize (8.40 t·ha^−1^), as well as SOC content (15.13 g·kg^−1^), storage (34.20 t·ha^−1^), and sequestration (7.11 t·ha^−1^). On the one hand, the higher cumulative carbon input under the A2B1 treatment explained the increased SOC sequestration and crop yield. On the other hand, the greater labile components of SOC (i.e., POC and LOC) and microbial necromass carbon (i.e., BNC, FNC, and MNC), mainly driven by improved stoichiometry, contributed to the crop yield gain and SOC sequestration, respectively, under A2B1 treatment. We emphasize that balanced fertilization is conducive to the sustainable production of wheat–maize planting systems in the North China Plain.

## Figures and Tables

**Table 1:** The SOC content, storage, and sequestration (ΔSOC storage) under different fertilization treatments.

Treatment		Bulk Density (g·cm^−3^)	SOC Content (g·kg^−1^)	SOC Storage (t·ha^−1^)	ΔSOC Storage (t·ha^−1^)
Initial		1.31	10.34	27.09	—
CK		1.29 ± 0.04 a	9.74 ± 0.80 d	25.16 ± 2.72 d	−1.93 ± 2.72 d
A1B1		1.24 ± 0.03 ab	11.94 ± 0.53 bc	29.59 ± 0.77 bc	2.50 ± 0.77 bc
A1B2		1.21 ± 0.04 bc	10.62 ± 0.37 cd	25.69 ± 0.92 cd	−1.40 ± 0.92 cd
A2B1		1.13 ± 0.03 d	15.13 ± 1.40 a	34.20 ± 3.26 a	7.11 ± 3.26 a
A2B2		1.16 ± 0.03 cd	13.59 ± 1.26 ab	31.51 ± 2.61 ab	4.41 ± 2.61 ab
ANOVA	A	19.69 **	28.69 ***	17.35 **	15.41 **
	B	0.01 ns	6.17 *	6.94 *	6.14 *
	A × B	2.77 ns	0.85 ns	0.23 ns	0.22 ns

The 3:0 distribution ratio of phosphorus (P) and potassium (K) fertilizers between the wheat and maize seasons combined with 450 kg·ha^−1^ of pure nitrogen (N) during the entire growth period of wheat and maize (A1B1). The 3:0 distribution ratio of P and K fertilizers between the wheat and maize seasons combined with 600 kg·ha^−1^ of pure N during the entire growth period of wheat and maize (A1B2). The 2:1 distribution ratio of P and K fertilizers between the wheat and maize seasons combined with 450 kg·ha^−1^ of pure N during the entire growth period of wheat and maize (A2B1). The 2:1 distribution ratio of P and K fertilizers between wheat and maize seasons combined with 600 kg·ha^−1^ of pure N during the entire growth period of wheat and maize (A2B2). No fertilization in the entire growth period of wheat and maize (CK). Different lowercase letters represented significant differences between different treatments based on Duncan’s multiple comparisons at *p* < 0.05. *, **, ***, and ‘ns’ indicate *p* < 0.05, *p* < 0.01, *p* < 0.001, and *p* > 0.05, respectively.

**Table 2:** Soil stoichiometry under different fertilization treatments.

Treatment		Stoichiometry					
		C:N	C:P	C:K	N:P	N:K	P:K
CK		10.54 ± 0.92 a	34.16 ± 0.93 b	1.82 ± 0.04 c	3.26 ± 0.33 bc	0.17 ± 0.01 c	0.05 ± 0.00 d
A1B1		9.32 ± 0.26 a	27.56 ± 1.49 c	1.91 ± 0.05 c	2.96 ± 0.08 cd	0.21 ± 0.00 b	0.07 ± 0.00 ab
A1B2		9.48 ± 0.79 a	25.28 ± 0.28 c	1.90 ± 0.02 c	2.68 ± 0.25 d	0.20 ± 0.01 b	0.08 ± 0.00 a
A2B1		9.21 ± 0.58 a	40.96 ± 0.86 a	2.44 ± 0.11 a	4.45 ± 0.20 a	0.26 ± 0.01 a	0.06 ± 0.00 cd
A2B2		9.47 ± 0.64 a	32.46 ± 2.07 b	2.10 ± 0.07 b	3.44 ± 0.31 b	0.22 ± 0.02 b	0.06 ± 0.01 bc
ANOVA	A	0.03 ns	173.04 ***	78.22 ***	75.72 ***	27.43 ***	24.50 **
	B	0.38 ns	47.54 ***	18.31 **	25.15 **	9.33 *	4.50 ns
	A × B	0.02 ns	15.83 **	16.28 **	8.12 *	4.76 ns	0.50 ns

Different lowercase letters represent significant differences between different treatments based on Duncan’s multiple comparisons at *p* < 0.05. *, **, ***, and ‘ns’ indicate *p* < 0.05, *p* < 0.01, *p* < 0.001, and *p* > 0.05, respectively.

**Table 3:** Fertilization period and fertilizer amount of “winter wheat–summer maize” crops under each treatment.

Fertilization Treatment	Fertilization Rate During Wheat Season (kg·ha^−1^)						Fertilization Rate During Maize Season (kg·ha^−1^)								
	Sowing Stage			Jointing Stage			Sowing Stage			Flare Opening Stage			Flowering-Filling Stage		
	N	P_2_O_5_	K_2_O	N	P_2_O_5_	K_2_O	N	P_2_O_5_	K_2_O	N	P_2_O_5_	K_2_O	N	P_2_O_5_	K_2_O
CK	0	0	0	0	0	0	0	0	0	0	0	0	0	0	0
A1B1	157.5	180	135	67.5	0	0	67.5	0	0	112.5	0	0	45	0	0
A1B2	210	180	135	90	0	0	90	0	0	150	0	0	60	0	0
A2B1	157.5	120	90	67.5	0	0	67.5	0	0	112.5	30	22.5	45	30	22.5
A2B2	210	120	90	90	0	0	90	0	0	150	30	22.5	60	30	22.5

## Data Availability

The data for this study are presented in the manuscript.
